# Self-organization in psychotherapy: testing the synergetic model of change processes

**DOI:** 10.3389/fpsyg.2014.01089

**Published:** 2014-10-02

**Authors:** Günter K. Schiepek, Igor Tominschek, Stephan Heinzel

**Affiliations:** ^1^Institute of Synergetics and Psychotherapy Research, Paracelsus Medical UniversitySalzburg, Austria; ^2^Day Treatment Center WestendMunich, Germany; ^3^Department of Psychology, Humboldt UniversityBerlin, Germany

**Keywords:** process-outcome research, self-organization, critical instability, sudden gains, obsessive compulsive disorder

## Abstract

In recent years, models have been developed that conceive psychotherapy as a self-organizing process of bio-psycho-social systems. These models originate from the theory of self-organization (Synergetics), from the theory of deterministic chaos, or from the approach of self-organized criticality. This process-outcome study examines several hypotheses mainly derived from Synergetics, including the assumption of discontinuous changes in psychotherapy (instead of linear incremental gains), the occurrence of critical instabilities in temporal proximity of pattern transitions, the hypothesis of necessary stable boundary conditions during destabilization processes, and of motivation to change playing the role of a control parameter for psychotherapeutic self-organization. Our study was realized at a day treatment center; 23 patients with obsessive compulsive disorder (OCD) were included. Client self-assessment was performed by an Internet-based process monitoring (referred to as the Synergetic Navigation System), whereby daily ratings were recorded through administering the Therapy Process Questionnaire (TPQ). The process measures of the study were extracted from the subscale dynamics (including the dynamic complexity of their time series) of the TPQ. The outcome criterion was measured by the Yale-Brown Obsessive Compulsive Scale (Y-BOCS) which was completed pre-post and on a bi-weekly schedule by all patients. A second outcome criterion was based on the symptom severity subscale of the TPQ. Results supported the hypothesis of discontinuous changes (pattern transitions), the occurrence of critical instabilities preparing pattern transitions, and of stable boundary conditions as prerequisites for such transitions, but not the assumption of motivation to change as a control parameter.

## Introduction

A large number of findings within psychotherapy research during recent years have revealed phenomena that, in classical medical models, would be considered anomalies. An example of such a phenomenon is the occurence of sudden gains in the course of symptoms, which go along with substantial changes in outcome criteria before the employment of any psychotherapeutic interventions (Ilardy and Craighead, 1999; Stiles et al., 2003; Tang et al., 2005, 2007; Vittengl et al., 2005; Busch et al., 2006; Kelly et al., 2007; Stulz et al., 2007). These findings suggest the psychotherapeutic process to be discontinuous and non-stationary instead of continuous and linear (Schiepek et al., 1992; Hayes and Strauss, 1998; Hayes et al., 2007; Schiepek and Perlitz, 2009; Haken and Schiepek, 2010). Together with findings that ascribe a comparatively small part of outcome variance to interventions and therapeutic techniques (Shapiro et al., 1994; Ahn and Wampold, 2001; Lambert and Ogles, 2004; Wampold, 2010), they give raise to substantial doubts on the classical view of linear proportionality between input (dosage) and output (outcome) in psychotherapy. Whereas input-output-mechanisms or mainstream dose-outcome models suppose some kind of linear or damped proportionalities between interventions and outcome, non-linear dynamic systems do not assume such proportionalities. Here small interventions can result in large effects on further system trajectories, or big interventions can be counterbalanced by the system dynamics—depending on the stability state of the system under consideration. By this, the mentioned results from the common factors research in psychotherapy point toward the non-linearity of therapeutic processes as a complex system, but further straightforward and positive indications of non-linear characteristics of change processes are necessary. Indeed, some studies produced findings of deterministic chaos, pattern formation and pattern transitions, non-linear precursors of critical events, and dynamic synchronization in high-resolution process markers of psychotherapy (e.g., Kowalik et al., 1997; Schiepek et al., 1997, 2009, in press; Tschacher et al., 1998, 2000; Granic et al., 2007; Ramseyer and Tschacher, 2008; Lichtwarck-Aschoff et al., 2012; Heinzel et al., 2014).

In order to grasp these phenomena, one has to entail models that do not ascribe psychotherapeutic effects merely to disorder-specific interventions and their appropriate dose. Instead, alternative explanations are being offered from the theory spectrum of complex systems, where changes are understood to result from self-organizing processes (e.g., Mahoney, 1991; Guastello, 1995; Hayes and Strauss, 1998; Orsucci, 2006; Pincus, 2009; Haken and Schiepek, 2010). Here, psychotherapy is conceptualized as a process that tries to support the conditions for self-organized change that underlies the enhancement of capacities of clients and client systems. Self-organization theories (Synergetics: Kelso, 1995; Haken, 2004; self-organized criticality: Bak et al., 1989; Bak, 1996; van Orden et al., 2003) make certain assumptions and provide models from which a number of hypotheses can be derived. Over the next few years the aim should be to design such models on how therapy works in a more explicit way (e.g., a mathematical formalism on common factor dynamics), but also to examine and to corroborate or falsify them.

Pattern formation and pattern transitions in dissipative systems are supposed to occur by so-called disequilibrium phase transitions that are neither linear nor incremental, but occur spontaneously and discontinuously (Kelso, 1995; Haken, 2004). In psychotherapy, these patterns refer to cognitive, affective, or interactional dynamics of clients in natural or clinical settings (e.g., client-therapist interaction). They have a certain organized complexity and at least a transient stability, which is to be seen and to be measured by the embedding of the systems trajectories in a state phase. The organized pattern of such trajectories in a phase space (e.g., the dimensions of this phase space are defined by the variables defining the system) is its *attractor*. In the study at hand, the system trajectories (time series representing the course of therapy) are drawn from daily internet-based self-assessments of the clients.

According to these models, change is a spontaneous process from within a non-linear system rather than a mere reaction to certain “interventions” from the outside. Hence, discontinuous order transitions can be expected that do not necessarily occur in reaction to any specific interventions. For an understanding of how psychotherapy works, the identification of cognitive-emotional patterns and pattern transitions will play an important role. Beyond this, we should proceed in explicit modeling of data-based common factors, their non-linear functions to each other, and the parameters mediating the interactions.

Pattern transitions (here we use the physical terms phase transitions or less specifically: order transitions) require a certain activity level of relevant control parameters which in many physical systems are related to an energy-flow through dissipative systems, thus forcing them out of an existing state of equilibrium. In psychotherapy we do not provide energies of any kind nor do we as therapists have any control parameters at our command. However, one could assume that intrinsic motivation for change, or other mental processes such as emotional involvement, activation of resources, or working intensity, contribute to a client's commitment toward change and can be supported by the therapist. These factors might thus be interpreted as the driving parameters of change in therapeutic processes (control parameter equivalents).

Another assumption is that spontaneous order changes are prepared and accompanied by critical fluctuations. Unlike catastrophe theory (Thom, 1976), this is a central prediction in Synergetics (Haken, 2004). In numerous physical experiments as well as in human development, critical fluctuations and processes related to deviation-amplifying feedback, but also processes of stabilizing changed dynamic patterns, require stable boundary conditions. In physical experiments, such conditions are provided through certain features of the experimental design; in humans they result from consistent experiences, in particular stable relationships and attachment to important others (Carter et al., 1997; Buchheim, 2011). This is where the key role of the client-therapist relationship enters the picture. A stable relationship between client and therapist yields the solid boundary conditions, which in turn allow for a destabilization (self-organized criticality) as well as a restabilization of processes.

To sum up, the hypotheses that were examined in the study at hand are the following:

1. For therapy effects to occur, stable boundary conditions are a necessary requirement; clients experience these in form of a positive atmosphere at the treatment facility and in positive therapeutic relationships. This hypothesis corresponds with current knowledge concerning the importance of good therapeutic relationships (Norcross, 2010) and stable emotional ties to attachment figures as prerequisites for learning processes (Carter et al., 1997). We expect a positive correlation between stability conditions like positive ward atmosphere or trustful working alliance at the one hand and therapy outcome at the other.
2. Phases of critical instability will occur in the course of psychotherapeutic processes. Such phases can be operationalized by local peaks (i.e., intensities that exceed the average level) of the dynamic complexity of change processes. Local maxima of dynamic complexity should be positively correlated with the therapy outcome (Schiepek et al., 2003; Haken and Schiepek, 2010; Gumz et al., 2012).
3. There is a necessity for an interaction between local critical instability and the stability of boundary conditions. (The stability of boundary conditions is operationalized by the experienced ward atmosphere and relationship with fellow patients.) Both are important and predictive for therapy results in as far as especially during instable periods of change processes experiences of stability are important (e.g., in the ward atmosphere or in the working alliance). Supposing a dialectic or counterbalancing relationship between both conditions of self-organization, a statistical interaction effect is expected.
4. Intrinsic motivation for change could be an equivalent of control parameters and is expected to positively correlate with therapy effects. In a strict sense, the effect of control parameters can only be tested in an experimental design which allows for a controlled linear increase of the parameter(s) (causally) related to an expected discontinuous phase transition. Since in human systems intra- or inter-individual conditions for change processes (parameters like motivation for change, intensity of emotions, stress level) usually are not available for external (experimental) control, we decide for an explorative and correlative approach.
5. Instationarities or order transitions are not dependent from specific interventions, therefore, we expect these transitions (sudden gains or losses) to occur independent of or already before major interventions are introduced. In the study at hand, the “major intervention” was exposure with response prevention (ERP) as part of a behavior therapy program for patients with obsessive compulsive disorder (OCD).
6. Changes occur in a discontinuous manner (hypothesis of instationarity), where the steepest change gradient is associated with the occurrence of critical instability. This hypothesis is directly based on the theoretical conjecture of Synergetics, that an enlargement of the potential valley of an existing pattern (attractor) implicates critical fluctuations and proceeds to a symmetry state with unavoidable symmetry breaking (transition to a changed pattern).

## Methods

### Setting and treatment

Twenty-three clients were recruited from a psychosomatic day treatment center in Munich, Germany, specialized in treating OCD. All participants gave informed consent to the inclusion into the study and participated on a deliberate base. The therapy rationale followed cognitive-behavioral therapy including psychoeducation, analysis of obsession- and compulsion-related behavior and cognitions, and exposure exercises (Lakatos and Reinecker, 2007). Therapy was primarily provided in a group setting, with one group session per day (Monday to Friday) accompanied by individual therapy sessions once or twice per week. The cognitive-behavioral therapeutic groups were guided by two experienced female therapists and two experienced female co-therapists (each had at least 10 years of practical experience in clinical settings). Additionally, clients participated in weekly relaxation training (Jacobson, 1990) and mentalization-focused sessions in a group setting.

From 23 clients, 18 participated in a period of massive exposure with response prevention (ERP) during their stay at the outpatient center. Five clients were not willing to participate in the massive ERP exercises and conducted minor exposure exercises and further cognitive therapy sessions instead. ERP exercises were conducted in individual therapy sessions after a period of preparation in the group setting like writing, signing an “ERP contract,” practicing coping skills, and performing minor exposure exercises. During the pre-ERP phase, the patients underwent exposure and coping exercises that focused on every-day and interpersonal situations of minor intensity and difficulty.

The outcome measurement by the Yale-Brown Obsessive Compulsive Scale (Y-BOCS) and the daily ratings by the Therapy Process Questionnaire (TPQ, see below) were realized by an Internet-based system (the Synergetic Navigation System, SNS) which was an integrative part of the therapy routine of the outpatient center in Munich. The SNS was used for continuously self-ratings of all patients (including diaries) applying a generic time schedule (here: process measures once per day, outcome measures two times per week). The system allows for graphical presentation and non-linear time series analysis of the process data.

A written informed consent was obtained from all participants after the procedures of the study had been fully explained.

### Participants

The sample covered 23 Caucasian clients diagnosed with OCD (for sample characteristics see Table 1). Clients were assessed by an experienced psychiatrist and classified in accordance with the International Classification of Diseases (ICD-10) as F42.0 “OCD, primarily obsessions and ruminations” (4 clients), as F42.1 “OCD, primarily compulsions” (4 clients), or as F42.2 “OCD, obsessions and compulsions” (15 clients). All clients completed the Y-BOCS biweekly and the TPQ once per day. The duration of treatment in days corresponds to the number of measurement points in the time series of TPQ ratings (mean: 60.2; *SD* = 12.7). Except where otherwise noted, all analyses were performed with the full sample of 23 clients.

**Table 1:** **Characteristics of the sample (*N* = 23)**.

	**Mean (*N* = 23**)	***SD***
Age	32.5	9.4
Male/Female	10/13	
Y-BOCS score pre	21.8	8.5
Y-BOCS score post	14.7	5.5
TPQ: symptom severity pre	4.5	1.3
TPQ: symptom severity post	3.7	0.9
Duration of treatment (days)	60.2	12.7

*The OCD symptom severity was obtained from the Yale-Brown Obsessive Compulsive Scale (Y-BOCS) and the Therapy Process Questionnaire (TPQ, scale II) before (pre) and after (post) therapy. A significant symptom reduction was reported in Y-BOCS scores [T_(22)_ = 6.26, p < 0.001; N = 23] and in TPQ symptom scores [T_(22)_ = 4.42, p < 0.001; N = 23]*.

### Measures and procedure

#### Process measures

Daily ratings were collected by an Internet-based device (Synergetic Navigation System, SNS, Schiepek, 2009). SNS is an ambulatory and real-time monitoring system which provides outcome- and especially process-assessment, with integrated mathematical tools for the analysis of non-linear and non-stationary time series (Schiepek and Perlitz, 2009; Schiepek and Aichhorn, 2013). Here we used a rating frequency of once per day and administered a questionnaire developed specifically for daily self-ratings during psychotherapeutic processes (TPQ) (Schiepek et al., 2003). The TPQ consisted of 47 items grouped into 5 scales. The TPQ allows for a reflection and assessment of emotions (like joy, fear, grief, anger, self-esteem), self-efficacy and therapeutic progress, hopefulness, working alliance, ward atmosphere, symptom severity, and other therapy-related experiences. The factorial structure of the TPQ is reported in Table 2 (for statistical details of the factor and item analysis see Schiepek et al., 2012).

**Table 2:** **Subscales of the Therapy Process Questionnaire (TPQ), modified for the application to outpatient centers**.

I Therapy progress (16.9% explained variance).
II Complaints and problem pressure/symptom severity (16.3% explained variance).
III Relationship quality and trust in therapists (16.3% explained variance).
IV Dysphoric affect (13.0% explained variance).
V Ward atmosphere and relationship with fellow patients (12.0% explained variance).

*The 47 items of the questionnaire are related to important common factors discussed in the literature (see Nischk et al., 2000 for a pre-study). The factor analysis of the TPQ was based on 149 in-patient therapy processes and resulted in a 5-factor solution, defining the 5 subscales of the questionnaire. Total explained variance is 74.5%. A confirmatory factor analysis performed for testing factor-model quality confirmed the 5-factor solution (for details see Schiepek et al., 2012)*.

The “control parameter” of the therapeutic change process was operationalized by the item “Today I was motivated to work on my problems and on their solution” of the TPQ (this item corresponds to the factor I “Therapy progress”).

The stable boundary conditions of the therapeutic destabilization process are represented by the experienced stability of the interpersonal environment of the patients. This experienced stability was measured by the overall mean of the factor V “Ward atmosphere and relationship with fellow patients” and factor III “Relationship quality and trust in therapists” of the TPQ. Both aspects of interpersonal stability, the relationship and working alliance with therapists and the relationship to other patients (ward atmosphere) were closely interrelated (*r* = 0.71, *p* < 0.001).

#### Identification of critical instabilities

The analysis of the time series concentrated on the dynamic complexity, which results from the product of a fluctuation measure *F* and a distribution measure *D*. The algorithm was designed to identify non-stationary phenomena and critical instabilities in short and coarse-grained time series. The fluctuation measure *F* is sensitive to the amplitude and frequency of changes in a time series, and the distribution measure *D* scans the scattering of values or system states realized within the range of possible values or system states (for technical details see Schiepek and Strunk, 2010; for clinical applications see Schiepek et al., 2003; Gumz et al., 2012; Heinzel et al., 2014). In order to identify non-stationarity, the combined measure is calculated within a data window of 7 measurement points, moving over the time series, resulting in a time series of dynamic complexity (C) for each item. Each value of this time series includes the information of 7 following days in the raw data. The movement of the running window goes from day to day and by this is partially overlapping.

It should be specified that this complexity measure is applicable to interval-scaled and regularly time-sampled real-world data without any further assumptions (e.g., concerning distribution characteristics, scale resolution, or length of time series). In practice, the length of the time series should be at least 20 measurement points, because this length of the time series is required to ensure sufficient validity of the measurement (Schiepek and Strunk, 2010) and because a change of complexity can only be measured within a sufficiently large period of data points. As other complexity measures like scaling exponents (*f^x^* noise, e.g., Pilgram and Kaplan, 1998), wavelet-based Time Frequency Distributions (e.g., Cohen, 1989; Lambertz et al., 2000), grammar complexity (e.g., Rapp et al., 1991), or fractal dimensionality (D2 Grassberger and Procaccia, 1983a,b or PD2 Skinner et al., 1994), dynamic complexity measures complexity from one of many possible points of view.

The self-organization model underlying this study is not focusing on each client's average level of complexity, but on the local peaks of complexity, indicating critical fluctuations and order transitions during the process. By this, not the averaged complexity, but the difference between the average and the maximum complexity of each item seems to be an appropriate indicator. The maxima result from local critical instabilities of the process. A phase of critical instability was defined as a sequence of days that contributes to a significant increase in dynamic complexity across the complete timeframe.

#### Outcome measures

The therapy outcome was identified by the self-rating form of the Y-BOCS (Goodman et al., 1989), which is world-wide the most commonly used rating scale for the intensity of obsessions and compulsions. It refers to the quality (e.g., concerning the experienced stress) as well as to the quantity (e.g., the duration of washing or checking rituals) of OCD symptoms. For the outcome assessment, we used the total score of the Y-BOCS combining the subscales “obsessions” and “compulsions.” The total score was transformed to a relative change score:

$$\text{relative YBOCS change}=\frac{post\;YBOCS-pre\;YBOCS}{pre\;YBOCS}\times \;100$$

For the representation of change gradients, the Y-BOCS was completed twice per week. For the sake of a comparison between the change scores of the patients, the Y-BOCS scores were z-transformed.

Another outcome measure was based on the relative change score of the factor II “Complaints and problem pressure” of the TPQ. The mean of the items representing this factor was calculated for the first week of treatment and then compared to the mean of the last week.

$$\text{relative symptom change }(\text{TPQ})=\frac{post\;symptoms-pre\;symptoms}{pre\;symptoms}\times \;100$$

The correlation between the relative Y-BOCS change and the TPQ-based relative symptom change was *r* = 0.62 (*p* = 0.002).

#### Data analysis

In order to test hypothesis 1, 2, and 4, correlations were calculated between factor scales of the TPQ and outcome measures (hypothesis 1 and 4), or between the difference of the mean and the maximum of the dynamic complexity score (maximum-mean complexity) and outcome measures (hypothesis 2). Further on, we tested hypotheses 1, 2, and 4 by hierarchical linear regression models predicting outcome measures from ward atmosphere, motivation to change, and maximum-mean complexity. The interaction hypothesis 3 (with reference to outcome measures) was tested by a 2 × 2 ANOVA with two factors based on a median split of all subjects with regard to the dimensions “high or low ward atmosphere” and “high or low maximum-mean complexity.” Hypotheses 5 and 6 were tested by *t*-tests of Y-BOCS and complexity mean levels in relation to the onset time of exposure with response prevention (ERP).

## Results

### Stability of boundary conditions (hypothesis 1)

The level of the TPQ scale V (“Ward atmosphere and relationship with fellow patients”) correlated with the relative Y-BOCS change (*r* = −0.49, *p* = 0.017) and with the relative symptom change (TPQ) (*r* = −0.54, *p* = 0.008). The relationship between the TPQ scale III (“Relationship quality and trust in therapists”) and the relative Y-BOCS change (*r* = −0.25, *p* = 0.251) did not meet the significance criterion. However, the relative symptom change (TPQ) correlated significantly with the “Relationship quality and trust in therapists” (*r* = −0.43, *p* = 0.042). The more stable the emotional and interpersonal boundary conditions of the therapeutic change processes, i.e., the better the quality of the therapeutic relationship and the ward atmosphere as experienced by the patients, the more the symptom severity (obsessions and compulsions) and problem pressure were reduced (as was expected in hypothesis 1).

### Intensity of critical instabilities (hypothesis 2)

The intensity of critical instabilities was represented by the difference between the mean and the maximum of the complexity of each therapy process, as explained above in the section Identification of critical instabilities. This criterion does not represent the overall complexity of the change dynamics (which can be interindividually different), but the local periods of order transition-related critical instabilities. It correlated non-significantly (*r* = −0.29, *p* = 0.177) with the relative Y-BOCS change. The relative symptom change (TPQ) was significantly related to the complexity score (*r* = −0.49; *p* = 0.018). The negative sign of the correlation coefficients means that an enhanced local complexity of the process corresponds to more reduced problem intensity or symptom severity after the psychotherapy. The results support hypothesis 2.

### Interactions between critical instability and stability of boundary conditions (hypothesis 3)

In order to assess interaction effects between “Ward atmosphere and relationship with fellow patients”) and maxium-mean complexity, four individual groups (maximum-mean complexity high vs. low; “Ward atmosphere and relationship with fellow patients” high vs. low) were formed per median split. Median splits were used as no a priori assumptions were made on criteria for “high” or “low” complexity and for “good” or “bad” ward athmosphere. A two [low (*N* = 12) vs. high complexity (*N* = 11)] by two [good (*N* = 12) vs. bad ward atmosphere (*N* = 12)] ANOVA was applied to investigate group differences in the two measures of therapy outcome. The median of the complexity score was found to be 0.097 and the median of the ward atmosphere was at 4.05.

**Figure 1:**
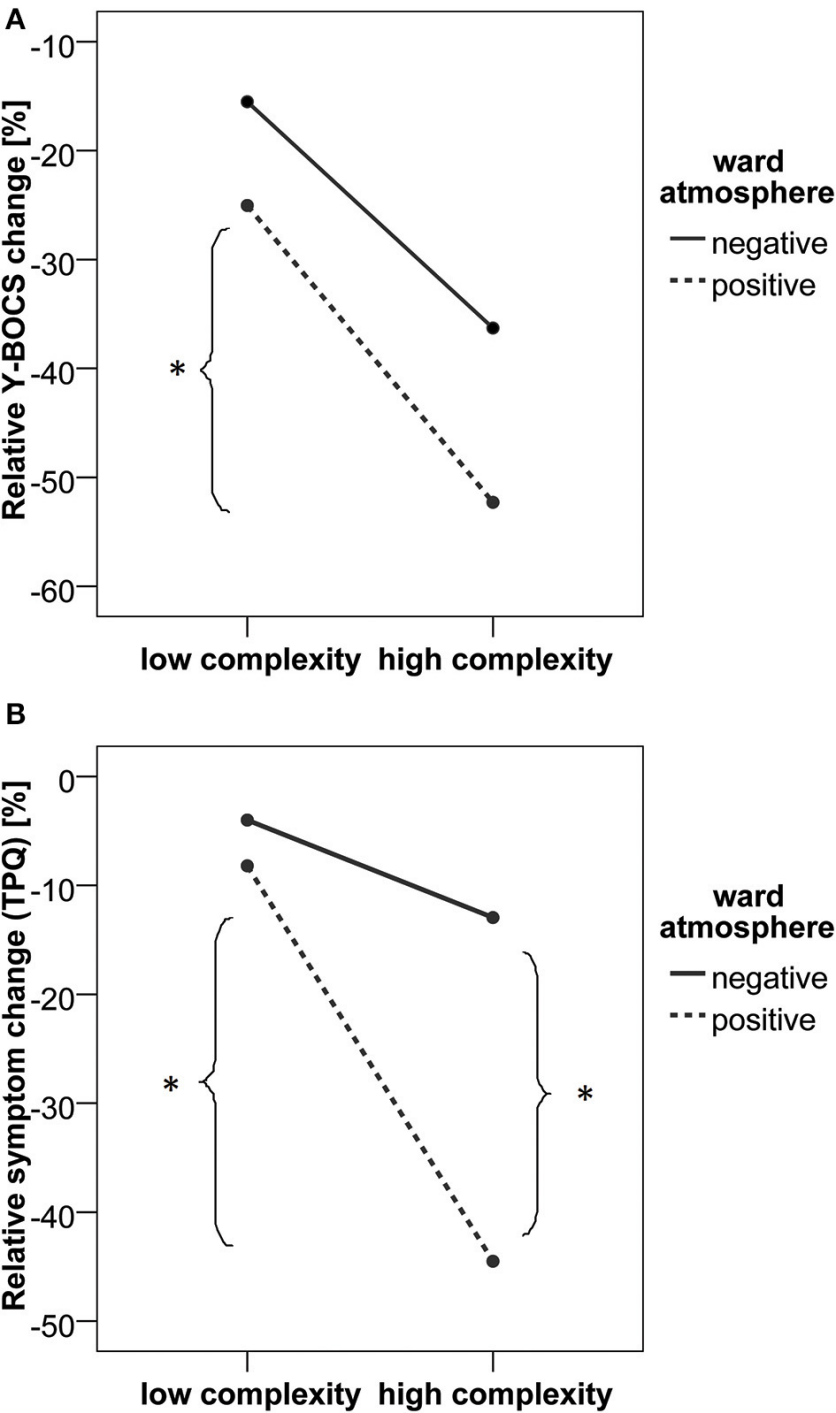
**Relative Y-BOCS change (A) and relative symptom change (TPQ, scale II) (B), related to the intensities of the local dynamic complexity (critical instability) of the change process and the ward atmosphere (stable boundary conditions of the change process)**. (^*^p < 0.05).

As shown in Figure 1A, the relative Y-BOCS change was greater in patients with higher local complexity scores [*F*_(1,19)_ = 8.92, *p* = 0.008, partial η^2^ = 0.319]. No significant main effect of the ward atmosphere was found [*F*_(1, 19)_ = 2.52, *p* = 0.129, partial η^2^ = 0.117]. When analyzing the group differences in relative Y-BOCS change, the complexity by ward atmosphere interaction was not significant [*F*_(1, 19)_ = 0.163, *p* = 0.691, partial η^2^ = 0.009]. However, *post-hoc t*-tests indicated that in patients who reported to experience a positive ward atmosphere, the ones who also went through at least one intensive phase of critical instability (high local complexity score and by this, high maximum-mean difference in complexity) had a higher relative Y-BOCS change compared to patients with low complexity scores [*t*_(10)_ = 2.29, *p* = 0.045, Cohen's *d* = 1.45].

The analysis of relative symptom change as measured by the TPQ revealed a significant complexity by ward atmosphere interaction [*F*_(1, 19)_ = 6.43, *p* = 0.020, partial η^2^ = 0.253]. As shown in Figure 1B, relative symptom change (TPQ) was greater in patients with higher local complexity scores [*F*_(1, 19)_ = 17.62, *p* < 0.001, partial η^2^ = 0.481] compared to patients with lower local complexity scores. Also, a positive ward atmosphere was related to a higher symptom change [TPQ, *F*_(1, 19)_ = 11.00, *p* = 0.004, partial η^2^ = 0.367]. *Post-hoc t*-tests showed that patients with high maximum-mean difference in complexity scores had higher relative symptom change (TPQ) only in the group of patients who reported a positive ward atmosphere [*t*_(10)_ = 4.60, *p* = 0.001, Cohen's *d* = 2.91]. A positive ward atmosphere was related to higher relative symptom change (TPQ) only in the high complexity group [*t*_(9)_ = 4.70, *p* = 0.001, Cohen's *d* = 3.13].

Taken together, the biggest therapy-related reduction of OCD symptoms was found within a group of patients that reported to experience a positive ward atmosphere and at least one intensive phase of critical instability (as was expected in hypothesis 3).

### Motivation to change (hypothesis 4)

Therapy outcome and motivation levels through the entire course of therapy showed no significant correlation (relative Y-BOCS change: *r* = −0.07, *p* = 0.736; relative symptom change (TPQ): *r* = −0.23, *p* = 0.282). Significant correlations were only found between the motivation level within the ERP phase and the relative symptom change (TPQ, *r* = −0.61, *p* = 0.007), but motivation during ERP phase was not related to relative Y-BOCS change (*r* = −0.15, *p* = 0.496). By this, hypothesis 4 was not or only partially confirmed. But there was a connection between mean patient motivation level and the TPQ subscale “Relationship quality and trust in therapists” (*r* = 0. 49, *p* = 0.018): A patient's high motivation for therapy is likely to facilitate the therapeutic relationship, and/or a trusting and stable working relationship will increase motivation for change.

### Outcome prediction by regression models (hypotheses 1, 2, and 4)

In two hierarchical linear regression models the predictors “Ward atmosphere and relationship with fellow patients” (ward atmosphere), motivation to change (motivation), intensity of local critical instabilities (complexity) were included block-wise into regression models to predict therapy outcome measured by relative Y-BOCS change (Table 3A) or by relative symptom change (TPQ, Table 3B). When predicting the relative Y-BOCS change, only the ward atmosphere contributed significantly to the model [*R*^2^ change = 0.244, *F* change_(1, 20)_ = 6.51, *p* = 0.019]. The full model, including motivation, ward atmosphere, and complexity, explained 29.9% of the variance in relative Y-BOCS change (see Table 3A). The regression to the relative symptom change (TPQ) showed that ward atmosphere [*R*^2^ change = 0.244, *F* change_(1, 20)_ = 6.95, *p* = 0.016] and complexity [*R*^2^ change = 0.145, *F* change_(1, 20)_ = 4.96, *p* = 0.038] significantly improved the model by explaining additional variance. The full model including motivation, ward atmosphere, and complexity explained 44.4% of the variance in relative symptom change (TPQ). These results corroborate the hypotheses 1, 2, and partially 4.

**Table 3:** **Hierarchical regression models**.

**Model**	**Included variables**	**β**	***T***	***P***	***R*^2^**	***R*^2^ change**	***F* change**	***P***
**(A)**								
1	Motivation	−0.074	−0.342	0.736	0.006	0.006	0.12	0.736
2	Motivation	0.081	0.398	0.695				
	Ward atmosphere	−0.518	−2.55	0.019	0.250	0.244	6.51	**0.019**
3	Motivation	0.142	0.686	0.502				
	Ward atmosphere	−0.488	−2.41	0.026				
	Complexity	−0.235	−1.16	0.262	0.299	0.049	1.34	0.262
**(B)**								
1	Motivation	−0.234	−1.10	0.282	0.055	0.055	1.22	0.282
2	Motivation	−0.079	−0.404	0.148				
	Ward atmosphere	−0.517	−2.58	0.016	0.299	0.244	6.95	**0.016**
3	Motivation	0.026	0.142	0.888				
	Ward atmosphere	−0.467	−2.58	0.018				
	Complexity	−0.403	−2.23	0.038	0.444	0.145	4.96	**0.038**

*Predictors: Motivation, ward atmosphere, local dynamic complexity (maximum-mean-difference of the dynamic complexity of all items of the TPQ). (A) Dependent variable: Relative Y-BOCS change. (B) Dependent variable: Relative symptom change (TPQ). Bold p-values are significant at p < 0.05*.

### Patterns of change (hypotheses 5 and 6)

**Figure 2:**
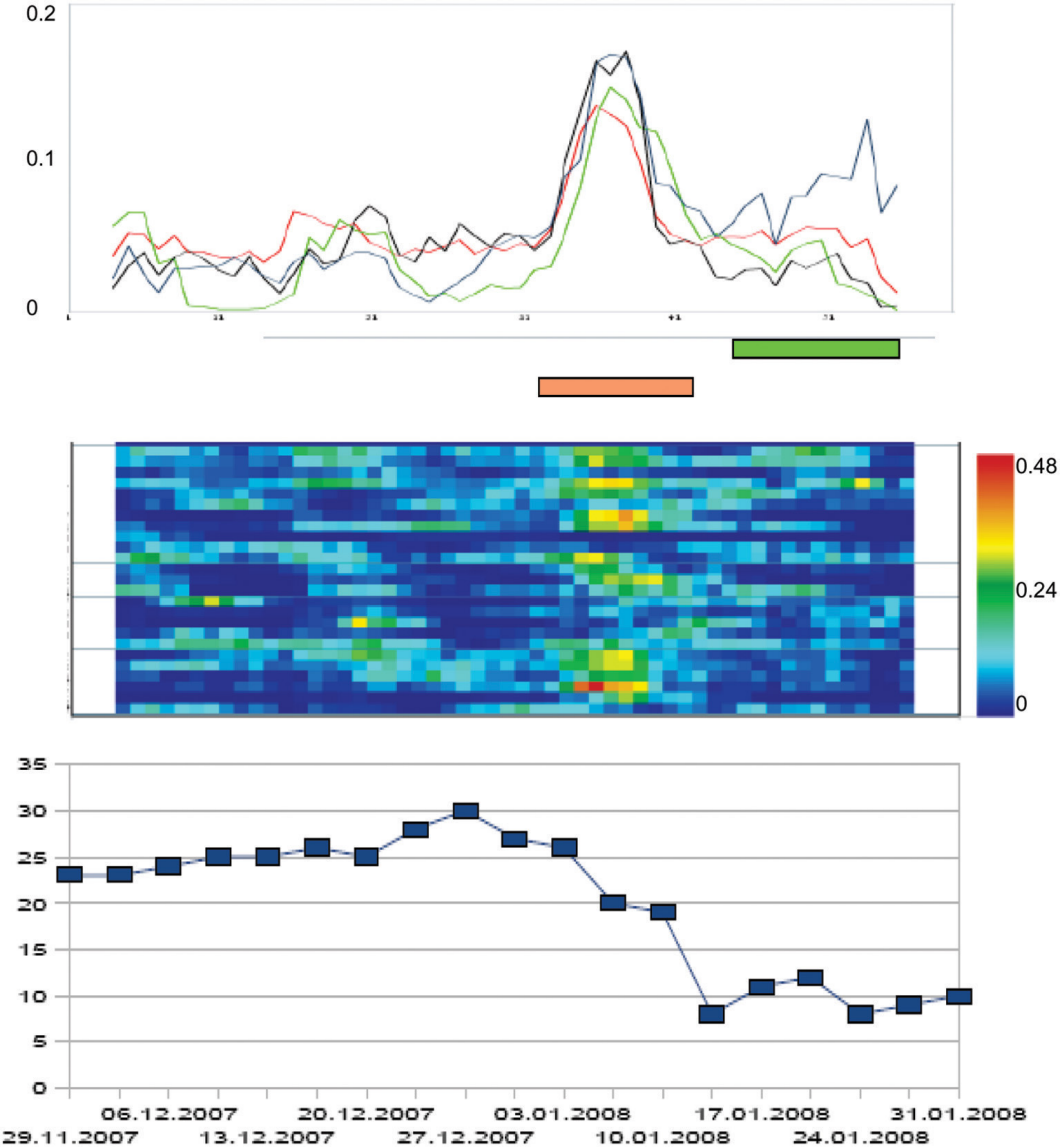
**Order transition during the therapy process of a patient with OCD**. The x-axis represents time, i.e., the duration (days) of the psychotherapy in a day treatment center (in this case: 64 days = measurement points). The curves at the top of the diagram represent the dynamic complexity of 4 subscales of the Therapy Process Questionnaire (TPQ): “Therapy progress” (blue), “Complaints and problem pressure” (black), “Dysphoric affects” (red), and “Getting new insights and perspectives” (green; this subscale corresponds to a factor from a former factor analysis of the questionnaire, see Haken and Schiepek, 2010). Dynamic complexity is calculated within an overlapping running window (width: 7 measurement points = days). In the middle part of the figure, the Complexity Resonance Diagram of the therapy process is represented. Each line of the diagram corresponds to an item of the chosen subscales of the TPQ. The dynamic complexity values of the time series of each item are translated into colors (yellow, orange, and red correspond to high complexity values). The lower part of the diagram represents the course of the Y-BOCS which was completed two times per week. The steepest gradient of symptom reduction was realized during the period of critical instability. Brown bar: period of statistically significant increased dynamic complexity. Green bar: Period of ERP.

The principal intervention of the cognitive-behavioral therapy applied to clients was exposure with response prevention (ERP). Consequently, we related the individual symptom severity trajectories to the onset of ERP. For each client, the individual ERP-onset was set at time point = 0, and the trajectories of the total Y-BOCS scores were related to this event. In 13 of the 18 patients which underwent ERP, the steepest gradient of symptom change was located *before* ERP-onset (compare hypothesis 5). Figure 2 represents this phase-transition-like phenomenon by the dynamics of a representative participant of our sample. If we calculate the mean of all individual trajectories of the ERP-subsample (*N* = 18), the effect is not abolished. The mean trajectory of the z-transformed individual total scores of the Y-BOCS has its steepest change gradient before ERP starts (time point = −4 days), and symptom severity reaches a significantly reduced level at the day of ERP onset at time point = 0 compared to the mean Y-BOCS level before the steepest change gradient [*t*_(17)_ = 3.07; *p* = 0.007].

The same procedure was accomplished with the mean dynamic complexity of all items of the TPQ, calculated within a moving window of 7 data points. This complexity dynamics was related to ERP-onset as well. When averaging the complexity curves of each individual treatment process, we can identify two distinct phases of critical instability on the group level. First, one can identify a clear-cut complexity peak at the beginning of treatment compared to the mean complexity outside of critical instabilities [*t*_(17)_ = 3.61, *p* = 0.004], which may be interpreted as an initial instability period representing individual doubts and varying degrees of working intensity at the start of the group process. Another clear-cut peak compared to mean complexity occurred 3 days before the steepest gradient of symptom reduction and about 7 days before the ERP-onset [*t*_(17)_ = 2.48, *p* = 0.026]. In terms of Synergetics, this corresponds to the assumed critical instabilities accompanying order transitions of a self-organizing system. The manifestation of complexity peaks at time-restricted windows of a change process is to be expected by the theory. This hypothesis could be confirmed by our data. The complexity peaks of each individual change process remain intact on group-level.

If we sum up the periods of significant critical complexities over all items (significance threshold *p* < 0.05) a frequency distribution results which itself can be examined for significant peaks. Compared to the mean relative frequency of critical instability over the whole process (*M* = 24.8%, *SD* = 13.5%), the relative frequency of critical instabilities is significantly increased at the beginning of the therapy [day −35 to –28, *M* = 42.6%, *SD* = 6.1%, *T*_(60)_ = 3.66, *p* = 0.001] and during a period of 11–4 days before ERP onset [*M* = 41.7%, *SD* = 4.2%, *T*_(60)_ = 3.48, *p* = 0.001, please refer to Heinzel et al. (2014) for a detailed description of the procedure]. The results corroborate hypotheses 5 and 6.

## Discussion

Conceptualizing psychotherapy as a self-organizing process leads to empirically testable hypotheses and thus appears to be inspiring for future research. In the current study, it was found that at least one period of increased complexity (critical instability) is necessary for effective treatment (hypothesis 2) and that temporally increased complexity is related to the steepest gradient of symptom reduction (hypotheses 5 and 6). In contrast to catastrophe theory, Synergetics predicts that critical fluctuations are an almost necessary precursor of phase transitions due to the broadening of the potential valley when the system dynamics approaches an instability point in non-linear systems and their far-from-equilibrium dynamics (Haken, 2004). At the same time, a positive ward atmosphere and relationship with fellow patients was beneficial for the outcome (hypothesis 1). This refers to the necessity of stable boundary conditions for pattern formation in complex systems. In fact, our results indicate that those patients who experience both, at least one phase of critical instability and a constantly positive ward atmosphere, reached the best psychotherapy outcome (hypothesis 3). These findings support the synergetic perspective on change dynamics as a process of destabilization within stable boundary conditions (Haken and Schiepek, 2010). However, no definite relationship between motivation and symptom change was revealed in the current study (hypothesis 4). When analyzing patterns of change in the course of psychotherapy processes, it was found that symptom change was temporally related to an increase in complexity (hypothesis 6). Interestingly, in many therapy processes the strongest change in symptoms already occurred before the application of ERP (hypothesis 5).

A recent fMRI-study (Schiepek et al., 2009, 2013) supported the results on order transitions in psychotherapy. Significant changes of brain activity patterns during order transitions were to be seen, whereas during periods without critical instabilities only marginal changes of brain activity took place. In this study, repeated fMRI scans were related to the degree of stability or instability of the ongoing dynamics. This was measured by the dynamic complexity of daily TPQ-ratings, and the maxima of these dynamics were used as an indicator of the most intensive fluctuation periods associated with discontinuous transition(s) during the therapies. Three or four scans were realized during each of the psychotherapy processes of 9 OCD patients and compared to the scans of 9 matched healthy controls without therapy.

Eight regions of interest were identified that are important in OCD-related neuronal processing: the anterior and medial cingulate cortex as well as the supplementary motor area (CC/SMA), the dorsolateral prefrontal cortex (DLPFC) right and left, the insula right and left, the parietal cortex right and left, and the cuneus. When interscan-intervals with order transitions in between were compared to intervals without order transitions, the changes of the number of significant voxels for the contrast between individualized symptom provoking pictures and neutral pictures showed increased BOLD responses during order transitions in all relevant brain regions. In healthy controls no significant changes in brain activity were found between the scans.

Both studies provide evidence in support of the hypothesis of a discontinuous change after destabilization of the psychotherapeutic process and indicate that activity patterns of neuronal and mental systems behave in a synchronized way. Changes were not found to occur gradually in the sense of a linear transition from the actual state to a targeted state and also do not appear to be a passive reaction to an applied intervention. Modeling psychotherapy as a cascade of order-to-order transitions (Haken and Schiepek, 2010) seems also to be a suitable explanation for the meanwhile large amount of data on sudden gains and on early rapid responses (e.g., Ilardy and Craighead, 1999; Stiles et al., 2003; Busch et al., 2006; Kelly et al., 2007; Stulz et al., 2007). In addition, the assumption can be supported that critical instabilities accompany the occurrence of therapeutic order transitions and that stable boundary conditions as experienced by clients are necessary for self-organizing dynamics. According to the present results, the least obvious causal factor in terms of therapy results is the motivation to change. This would have been expected since control parameters are important conditions for order transitions, as theory states, and human change processes are driven by conditions generated within the system, i.e., the client. This might well-fit the important contribution of client variables to therapy success (e.g., Bohart and Tallman, 2010).

### Limitations

One weakness of our study was operationalizing motivation to change as a control parameter equivalent by using only one TPQ item. Measurements based on only one item or one variable are on insecure footing. There are other change driving parameters which support patients' development besides their intrinsic motivation for change, such as level of suffering, activation of resources, experiencing self-efficacy, or therapeutic success. As mentioned in the introduction, a correlational approach generally allows only for a weak operationalization of what is meant by the control parameter concept. A control parameter in its specific sense, like temperature gradients for the emergence of convection streams in fluids, would need a gradual increase in its intensity and then be accompanied by an order transition at a specific threshold of the parameter value. In the case of psychotherapy this would ask for an experimental design for intrinsic variables which reaches its limitations by logical, ethical, and practical reasons. Another limitation of the study is the small number of subjects. However, with a small number of subjects, effects need to be relatively strong to be recognized as significant. A reexamination of the present hypotheses is in progress with a considerably larger sample from different clinical settings, diagnoses, and therapy concepts.

### Perspectives

The synergetic model of human change processes would provide potential for the integration of the results on common factors (Duncan et al., 2010; Wampold, 2010), and furthermore, for an integration of the medical and the common factors model of psychotherapy. The only requirement and precondition would be that the medical model is not restricted to a linear model. Both—the medical model as well as the common factors model—can be subsumed under the following assumptions:

1. Psychotherapy consists in supporting self-organization processes.
2. Most common factors are conditions for self-organizing processes (they can be subsumed to the concept of “generic principles,” see Haken and Schiepek, 2010) and thus they are “specific” in the sense of “theoretically founded.”
3. There is no linear input-output mechanism of therapeutic actions (techniques).
4. An understanding and a specific modeling of intra-systemic mechanisms of systems re-organization is possible, as was shown for the neuronal mechanisms of self-organized de-synchronization (coordinated reset) of pathologically oversynchronized neuronal systems underlying Parkinsonian Disease or Tinnitus by Tass and coworkers (e.g., Tass and Hauptmann, 2007).

Another reason for the possible integration of the common factors model (Wampold, 2010) into the self-organization model is that mental and brain dynamics follow the same principles of self-organization (Kelso, 1995; Haken, 2002; Orsucci, 2006; Tass and Hauptmann, 2007; Deco et al., 2011), thereby allowing for an integrated psychological and neurobiological psychotherapy (Grawe, 2004).

Another consequence of this approach might be that non-linear features such as order transitions or critical instabilities can be measured and analyzed during ongoing psychotherapy processes, fed back immediately, and used for adaptive therapy planning, which justifies the application of real-time monitoring systems in psychotherapy (Lambert, 2010; Schiepek and Aichhorn, 2013).

We realized this real-time monitoring approach in several psychotherapy hospitals (Inpatient Treatment Center and Day Treatment Center of the Christian Doppler Clinic Salzburg, Austria; Systelios Health Center Siedelsbrunn, Germany; Psychosomatic Clinic Bad Zwischenahn, Germany; Day Treatment Center Munich, Germany; Fachklinik Hirtenstein, Oberstorf, Germany) and by this, we got 647 data sets from patients with different diagnoses (Table 4). More than 120 cases have a time series length of more than 100 measurement points. This might be one of the largest data sets with equidistant (daily) self-ratings available in psychotherapy research (mean: 73.4 measurement points, 3.0% missing data), which allows for detailed non-linear time series analyses and validation of theoretical modeling. As can be demonstrated, the patterns of change visualized and analyzed by such kind of high frequency measures are quite different from the time series we get from session-by-session ratings: non-linearity, chaos, and non-stationarity (pattern transitions) are quite evident. By this the Synergetic Navigation System offers new perspectives of data mining in routine practice, online data analysis (methods as Dynamic Complexity, Recurrence Plots, Complexity Resonance Diagrams, Permutation Entropy, or Correlation Pattern Analysis are integrated in the SNS), and therapy feedback (Schiepek et al., 2014).

**Table 4:** **The data set resulting from several years of application of an internet-based device, the Synergetic Navigation System, at 6 psychotherapeutic hospitals or day treatment centers**.

**Diagnosis**	***N***	**% Women**	**Measurement points [days] (*SD*)**	**% Missings (*SD*)**	**Age (*SD*)**
f1: psychoactive substance use	49	2	85.6 (27.3)	4.5 (4.04)	46.7 (10.0)
f2: delusional disorders	11	37	88.9 (31.3)	3.7 (4.1)	28.2 (7.8)
f3: mood disorders	299	55	67.4 (33.4)	2.9 (3.55)	44.2 (10.8)
f4: stress and somatoform disorders	172	69	72.3 (47.6)	2.8 (3.43)	38.3 (12.1)
f5: physiological disturbance	8	88	61.4 (23.5)	4.9 (4.35)	26.3 (6.6)
f6: personality disorders	107	73	86.4 (44.0)	2.4 (3.39)	33.3 (11.0)
Total	647	43	73.4 (39.7)	3.0 (3.6)	40.6 (12.1)

*N = 647 cases, distributed over different diagnoses. Daily self-ratings are done by the Therapy Process Questionnaire or some setting-specific modifications of this questionnaire*.

One important aspect of continuous feedback could be the identification of precursors of critical instabilities—not only in psychotherapy or consulting, but also in suicide prevention. By this an early warning system could be developed for critical events (like suicidal attempts) in non-linear chaotic systems (like human beings) which cannot be predicted on the long run (Schiepek et al., 2011). Indicators of critical events could be locally increased dynamic complexity or other complexity markers, increased linear correlation or non-linear synchronization (e.g., Transinformation) between components, subsystems, or different dynamic aspects of the system, increased autocorrelation of the dynamics, increased coupling of system components or subsystems (e.g., Pointwise Conditional Coupling Divergence), or transition markers in Recurrence Plots (Orsucci et al., 2006; Haken and Schiepek, 2010; Schiepek et al., 2011; Dakos et al., 2012; Lichtwarck-Aschoff et al., 2012). Interestingly, Lichtwarck-Aschoff et al. (2012) used Recurrent Quantification Analysis (Orsucci et al., 2006; Webber et al., 2009) and the entropy markers of this quantification in order to identify critical instabilities in therapeutic change processes of mother-child interaction dynamics. Recurrence Plots seem to be a very useful instrument for the visualization and quantification of critical instabilities and order transitions in human change processes. Besides methods based on dynamic complexity (like Complexity Resonance Diagrams) also Recurrence Plots are available in the SNS for routine application in therapy monitoring. Actually converging methods for the identification of discontinuous dynamics seem to be available which should be integrated and/or systematically tested against each other in order to develop a deeper understanding of critical transitions in human systems.

### Conflict of interest statement

The authors declare that the research was conducted in the absence of any commercial or financial relationships that could be construed as a potential conflict of interest.
